# Serum syndecan1 has the potential to reflect activity at diagnosis and predict death during follow-up in patients with ANCA-associated vasculitis

**DOI:** 10.1186/s13075-024-03393-8

**Published:** 2024-09-20

**Authors:** Taejun Yoon, Jang Woo Ha, Jung Yoon Pyo, Eunhee Ko, Sung Soo Ahn, Jason Jungsik Song, Yong-Beom Park, Sang-Won Lee

**Affiliations:** 1 Department of Medical Science, BK21 Plus Project, College of Medicine, Yonsei University, Seoul, Republic of Korea; 2 Division of Rheumatology, Department of Internal Medicine, Yongin Severance Hospital, Yonsei University College of Medicine, Yongin, Gyeonggi-do Republic of Korea; 3 Department of Rheumatology, Seoul National University Bundang Hospital, Seongnam, Republic of Korea; 4 Division of Rheumatology, Department of Internal Medicine, Severance Hospital, Yonsei University College of Medicine, 50-1 Yonsei-ro, Seodaemun-gu, 03722 Seoul, Republic of Korea; 5 Institute for Immunology and Immunological Diseases, Yonsei University College of Medicine, Seoul, Republic of Korea

**Keywords:** Syndecan1, Antineutrophil cytoplasmic antibody, Vasculitis, Activity, Mortality

## Abstract

**Objectives:**

This study investigated whether serum syndecan1 at diagnosis reflects activity at diagnosis and predicts poor outcomes during follow-up in patients with antineutrophil cytoplasmic antibody (ANCA)-associated vasculitis (AAV).

**Methods:**

The study included 79 patients with AAV from the cohort of Korean patients diagnosed with AAV. AAV-specific indices, including the Birmingham vasculitis activity score (BVAS), five-factor score (FFS), 36-item short-form survey (SF-36) physical and mental component summary (PCS and MCS), and vasculitis damage index (VDI), were assessed. Laboratory data including erythrocyte sedimentation rate (ESR) and C-reactive protein (CRP) levels were also collected. The highest tertile and upper half of the BVAS were tentatively defined as having high AAV activity. Serum syndecan1 levels were measured in sera stored at diagnosis.

**Results:**

Serum syndecan1 at diagnosis was significantly correlated with AAV activity and functional status, as assessed by BVAS, FFS, SF-36 PCS, MCS, and acute-phase reactants, including ESR and CRP. Patients with serum syndecan1 ≥ 76.1 ng/mL at diagnosis, and those with serum syndecan1 ≥ 60.0 ng/mL at diagnosis showed significantly higher risks for the highest tertile and the upper half of BVAS at diagnosis than those without, respectively. Patients with serum syndecan1 ≥ 120.1 ng/mL at diagnosis had a significantly higher risk for all-cause mortality during follow-up than those without, and further, exhibited a significantly lower cumulative patients’ survival rate than those without.

**Conclusion:**

Serum syndecan1 at diagnosis may not only reflect AAV activity at diagnosis but may also be associated with all-cause mortality during follow-up.

**Supplementary Information:**

The online version contains supplementary material available at 10.1186/s13075-024-03393-8.

## Background

Syndecans are proteins that constitute a family of heparan sulphate proteoglycans comprising four members: syndecan1, 2, 3, and 4, of which syndecan1 and 3 carry chondroitin sulphate. All four syndecans contain three domains: an ectodomain (N-terminal extracellular domain), a transmembrane domain, and a cytoplasmic domain (C-terminal intracellular domain) [1]. Of the four syndecans, syndecan1 is highly expressed in fibroblastic and epithelial cells compared to endothelial cells, and its expression is known to be increased in the skin, liver, kidney, and lung tissues [2]. Syndecan1 can be released from cells expressing syndecan1 on the surface via cleavage by proteinases such as trypsin. Circulating syndecan1 may bind to a proliferation-inducing ligand (APRIL) and aggregate, leading to enhanced binding affinity to the transmembrane activator, calcium-modulator, and cyclophilin ligand interactor (TACI) or B-cell maturation antigen (BCMA) of B cells. Consequently, circulating syndecan1 may increase B cell survival and accelerate their differentiation into antibody-producing plasma cells. Therefore, circulating syndecan1 may not only have the potential to aggravate autoantibody-associated diseases, such as systemic lupus erythematosus and monoclonal gammopathy, but also act as a biomarker reflecting activity and predicting prognosis in each disease [3, 4]. Another study has also shown that serum syndecan-1 levels may reflect vascular endothelium injury and mucosal damage in IgA vasculitis [5].

Antineutrophil cytoplasmic antibody (ANCA)-associated vasculitis (AAV) is a group of small-vessel vasculitis with few or no immune deposits [6, 7] and has three subtypes according to the typical manifestations of microscopic polyangiitis (MPA), granulomatosis with polyangiitis (GPA), and eosinophilic GPA (EGPA) [8–10]. ANCA is an autoantibody that recognises cytoplasmic autoantigens released by primed neutrophils, myeloperoxidase (MPO), and proteinase 3 (PR3). Circulating ANCA may bind to primed neutrophils, mostly forming neutrophil dimers and initiating ANCA-mediated neutrophil activation [11]. Given the role of circulating autoreactive ANCA in the pathogenesis of AAV and the effect of circulating syndecan1 on B cell activation, it can be reasonably speculated that circulating syndecan1 may be positively correlated with cross-sectional activity and significantly associated with fatal complications during the disease course of AAV. However, till date, the clinical importance of serum syndecan1 in AAV has not been investigated. Hence, in the present study, we investigated whether serum syndecan1 at diagnosis could reflect activity at diagnosis and predict poor outcomes during follow-up in patients with AAV.

## Materials and methods

### Patients

In the present study, 80 patients were randomly selected from a cohort of Korean patients with AAV. This was a prospective and observational cohort study of AAV initiated and conducted in this hospital. The inclusion criteria were (i) the first classification of AAV at the Division of Rheumatology, Department of Internal Medicine, Yonsei University College of Medicine, from 2016 to 2023; (ii) the fulfilment of the following criteria and definitions: the algorithm for AAV proposed by the European Medicine Agency in 2007 (the 2007 EMA algorithm), and the revised International Chapel Hill Consensus Conference Nomenclature of Vasculitides suggested in 2012 (the 2012 CHCC definition) [6, 7]; (iii) the reclassification of AAV according to the new classification criteria for MPA, GPA, and EGPA, proposed by the American College of Rheumatology and European Alliance of Associations for Rheumatology in 2022 (the 2022 ACR/EULAR criteria) [8–10]; (iv) the equipped medical records containing clinical and laboratory data sufficient for classifying AAV, assessing activity, and identifying poor prognosis from the diagnosis to the last visit; (v) the follow-up duration for at least six months or greater; (vi) the presence of the consent form for providing clinical data as well as blood samples at diagnosis; (vii) the presence of the 36-item short-form survey (SF-36) physical and mental component summary (PCS and MCS) completed by the patients [12]; (viii) the absence of serious medical conditions mimicking AAV at diagnosis such as severe infectious and cancerous diseases [8–10]; (ix) the absence of medical or drug history affecting ANCA positive such as primary sclerosing cholangitis or propylthiouracil [13, 14]; and (x) the absence of exposure to moderate to high doses of glucocorticoids or immunosuppressive drugs for AAV treatment within four weeks before diagnosis. Of the 80 patients, one was excluded because the condition of the stored serum was not available, and 79 were finally analysed in this study.

### Clinical and laboratory data

In terms of variables at the time of AAV diagnosis, age, sex, ex-smoker status, and body mass index were collected as demographic data. Positive results of not only MPO-ANCA and PR3-ANCA measure by an immunoassay but also perinuclear (P)-ANCA and cytoplasmic (C)-ANCA detected by an indirect immunofluorescence assay were considered ANCA positive in this study according to the 2022 ACR/EULAR criteria for AAV [8–10, 15]. AAV-specific indices included the Birmingham vasculitis activity score (BVAS), the five-factor score (FFS), SF-36 PCS and MCS, and the vasculitis damage index (VDI) were assessed [12, 16–18]. Type 2 diabetes mellitus, hypertension, and dyslipidaemia were reviewed as comorbidities [19]. Laboratory data including erythrocyte sedimentation rate (ESR) and C-reactive protein (CRP) levels were also collected. Poor outcomes of AAV and medications, including glucocorticoids and immunosuppressive drugs, were evaluated during follow-up.

### Poor outcomes

Poor AAV outcomes were defined as all-cause mortality and end-stage kidney disease after AAV diagnosis. The follow-up duration based on each poor outcome was defined as the period from diagnosis to its occurrence in patients with a corresponding poor outcome, whereas the duration from diagnosis to the last visit was defined for those without.

### High activity of AAV

In this study, the highest tertile and upper half of the BVAS were tentatively defined as having high AAV activity and were subjected to statistical analyses.

### Blood sampling

On the day AAV was classified and AAV-specific indices regarding activity, function, and major organ damage were assessed, whole blood was obtained from patients with AAV. Sera was immediately isolated from whole blood and stored at -80℃.

### Measurement of serum syndecan1

Serum syndecan1 levels were measured using enzyme-linked immunosorbent assay kits (Abcam, Cambridge, UK) from collected and stored sera at diagnosis.

### Statistical analyses

All statistical analyses were performed using IBM SPSS Statistics for Windows version 26 (IBM Corp., Armonk, NY, USA). Continuous and categorical variables were expressed as medians (25 − 75 percentiles) and numbers (percentages). The correlation coefficient (r) between the two variables was obtained using Pearson and Spearman correlation analysis. The significant area under the curve (AUC) was confirmed by receiver operator characteristic (ROC) curve analysis. The optimal cutoff was extrapolated by performing ROC curve analysis and selected as that with the maximum sum of sensitivity and specificity. The relative risk (RR) of the cutoff for all-cause mortality was analysed using contingency tables and the chi-square test. The cumulative survival rates between the two groups were compared using Kaplan-Meier survival analysis with the log-rank test. The multivariate Cox hazard model using variables with statistical significance in the univariate Cox hazard model was used to obtain hazard ratios (HRs) during a considerable follow-up duration. Statistical significance was set at *P* < 0.05.

## Results

### Characteristics of patients

In terms of variables at diagnosis, the median age of the 79 patients was 64.0 years, and 40.5% and 59.5% of the patients were men and women, respectively. Thirty-eight, 24, and 17 patients were diagnosed with MPA, GPA, or EGPA, respectively. MPO-ANCA (or P-ANCA) and PR3-ANCA (or C-ANCA) were positive in 44 (55.7%) and 12 (15.2%) patients, respectively. The median BVAS, FFS, SF-36 PCS and MCS, and VDI were 5.0. 0, 52.5, 54.9, and 3.0, respectively. Among the organ involvements, the most common were lung involvement with 50 cases (63.3%), ear/nose/throat involvement with 41 cases (51.9%), and kidney involvement with 38 cases (48.1%). Of the 79 patients, 17, 25, and 14 had type 2 diabetes mellitus, hypertension, and dyslipidaemia, respectively. The median ESR and CRP were 21.0 mm/h and 3.6 mg/L, and serum syndecan1 was measured at 52.2 ng/mL as a median value. In terms of variables during follow-up, of the 79 patients, six (7.6%) died and 18 (22.8%) experienced progression to ESKD for the median follow-up durations based on each poor outcome of 26.7, and 26.3 months, respectively.

Of the 79 patients, 78 received glucocorticoids, and the most commonly administered immunosuppressive drug was cyclophosphamide, followed by azathioprine (Table 1).

**Table 1:** Characteristics of patients with AAV at diagnosis and during follow-up (*N* = 79)

Variables	Values
**At the time of diagnosis**	
**Demographic data**	
Age (years)	64.0 (52.0 − 74.0)
Male sex (N, (%))	32 (40.5)
Female sex (N, (%))	47 (59.5)
Ex-smoker (N, (%))	3 (3.8)
Body mass index (kg/m^2^)	22.4 (20.8 − 24.7)
**AAV subtype (N**,** (%))**	
MPA	38 (48.1)
GPA	24 (30.4)
EGPA	17 (21.5)
**ANCA type and positivity (N**,** (%))**	
MPO-ANCA (or P-ANCA) positive	44 (55.7)
PR3-ANCA (or C-ANCA) positive	12 (15.2)
**AAV-specific indices**	
BVAS	5.0 (3.0 − 17.0)
FFS	0 (0 − 1.0)
SF-36 PCS	52.5 (34.4 − 67.8)
SF-36 MCS	54.9 (39.7 − 71.9)
VDI	3.0 (2.0 − 4.0)
**Organ involvement**	
Constitutional symptoms	17 (21.5)
Skin	12 (15.2)
Eyes/mucosal	7 (8.9)
Ear/nose/throat	41 (51.9)
Lung	50 (63.3)
Nervous	27 (34.2)
Cardiovascular	9 (11.4)
Abdominal	0 (0)
Kidney	38 (48.1)
Renal biopsy	31 (39.2)
**Comorbidities (N**,** (%))**	
Type 2 diabetes mellitus	17 (21.5)
Hypertension	25 (31.6)
Dyslipidaemia	14 (17.7)
**Acute-phase reactants**	
ESR (mm/hr)	21.0 (7.0 − 74.8)
CRP (mg/L)	3.6 (0.9 − 28.6)
**Laboratory results**	
White blood cell count (/mm^3^)	7,610.0 (5,960.0 − 10,560.0)
Haemoglobin (g/dL)	12.4 (10.2 − 13.6)
Platelet count (x1,000/mm^3^)	247.0 (192.3 − 362.0)
Blood urea nitrogen (mg/dL)	19.2 (13.8 − 28.7)
Serum creatinine (mg/dL)	0.8 (0.6 − 1.6)
Total serum protein (g/dL)	6.8 (6.3 − 7.3)
Serum albumin (g/dL)	4.2 (3.6 − 4.4)
C3 (mg/dL)	113.5 (97.5 − 126.3)
C4 (mg/dL)	25.4 (20.2 − 31.0)
**Serum syndecan1 (ng/mL)**	52.2 (29.3 − 85.5)
**During follow-up**	
**Poor outcome (N**,** (%)**	
All-cause mortality	6 (7.6)
ESKD	18 (22.8)
**Follow-up duration based on each poor outcome (months)**	
All-cause mortality	26.7 (12.1 − 45.7)
ESKD	26.3 (9.0 − 45.7)
**Medications**	
Glucocorticoids	78 (98.7)
**Induction therapy**	
Cyclophosphamide	52 (65.8)
Rituximab	16 (20.3)
**Maintenance therapy**	
Rituximab	5 (6.3)
Mycophenolate mofetil	20 (25.3)
Azathioprine	47 (59.5)
Tacrolimus	7 (8.9)
Methotrexate	3 (3.8)

Values are expressed as a median (25 − 75 percentile) or N (%)

*ANCA* antineutrophil cytoplasmic antibody, *AAV* ANCA-associated vasculitis, *MPA* microscopic polyangiitis, *GPA* granulomatosis with polyangiitis, *MPO* myeloperoxidase, *P* perinuclear, *PR3* proteinase 3, *C* cytoplasmic, *BVAS* the Birmingham vasculitis activity score, *FFS* the five-factor score, *SF36* 36-item short form survey, *PCS* physical component summary, *MCS* mental component summary, *VDI* vasculitis damage index, *ESR* erythrocyte sedimentation rate, *CRP* C-reactive protein, *C3* complement 3, *C4* complement 4, *ESKD* end-stage kidney disease

Serum syndecan1 levels for all patients with MPA, GPA, and EGPA are presented in the Supplementary Fig. 1. The range of syndecan1 levels was from 14.97 to 1504 ng/mL.

### Correlation of variables with serum syndecan1 at diagnosis

Serum syndecan1 at diagnosis was significantly correlated with BVAS (*r* = 0.364), FFS (*r* = 0.400), all-cause mortality (*r* = 0.291), ESR (*r* = 0.505), CRP (*r* = 0.286), white blood cell count (*r* = 0.353), blood urea nitrogen (*r* = 0.467), and serum creatinine (*r* = 0.397). Meanwhile, serum syndecan1 at diagnosis was inversely correlated with SF-36 PCS (*r* = − 0.373), SF-36 MCS (*r* = − 0.330), haemoglobin (*r* = − 0.405), and serum albumin (*r* = − 0.451) at diagnosis (Table 2).

**Table 2:** Correlation analysis of continuous variables for serum syndecan1 levels at diagnosis in patients with AAV (*N* = 79)

Variables	Univariable	
	Beta	*P* value
**Demographic data**		
Age (years)	0.119	0.298
Body mass index (kg/m2)	−0.008	0.942
**AAV-specific indices**		
BVAS	0.364	0.001
FFS	0.400	< 0.001
SF-36 PCS	−0.373	0.001
SF-36 MCS	−0.330	0.003
VDI	0.149	0.197
**Poor outcome**		
All-cause mortality	0.291	0.009
ESKD	0.173	0.127
**Acute-phase reactants**		
ESR (mm/hr)	0.505	< 0.001
CRP (mg/L)	0.286	0.013
**Laboratory results**		
White blood cell count (/mm^3^)	0.353	0.001
Haemoglobin (g/dL)	−0.405	< 0.001
Platelet count (x1,000/mm^3^)	−0.066	0.563
Blood urea nitrogen (mg/dL)	0.467	< 0.001
Serum creatinine (mg/dL)	0.397	< 0.001
Total serum protein (g/dL)	0.045	0.701
Serum albumin (g/dL)	−0.451	< 0.001
C3 (mg/dL)	0.118	0.314
C4 (mg/dL)	0.061	0.601

*ANCA* antineutrophil cytoplasmic antibody, *AAV* ANCA-associated vasculitis, *CI* confidence interval, *BVAS* the Birmingham vasculitis activity score, *FFS* the five-factor score, *SF36* 36-item short form survey, *PCS* physical component summary, *MCS* mental component summary, *VDI* vasculitis damage index, *ESR* erythrocyte sedimentation rate, *CRP* C-reactive protein, *C3* complement 3, *C4* complement 4

### Relative risks of cut-off of serum syndecan1 for high activity of AAV at diagnosis

**Fig. 1:**
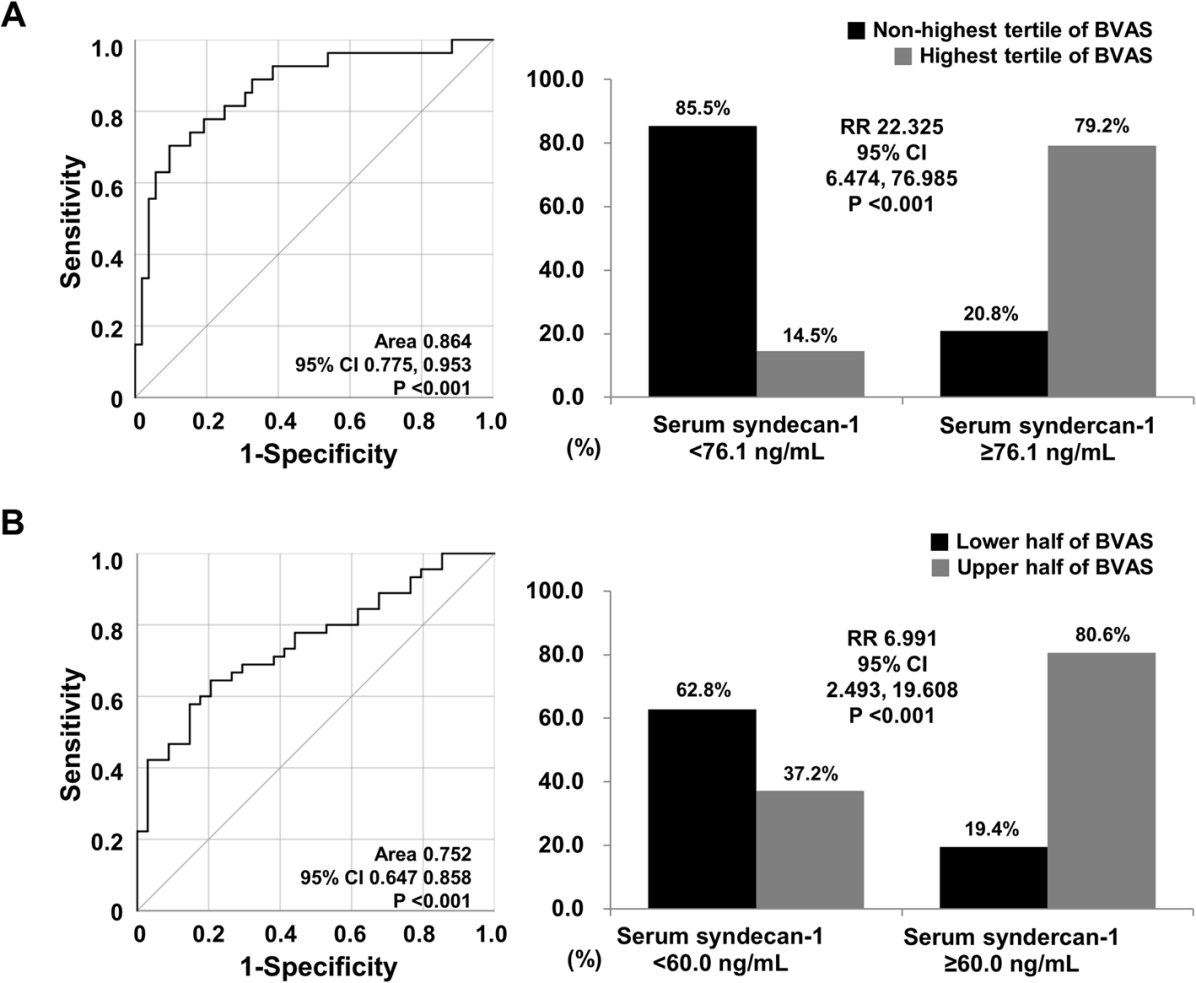
Optimal cut-off and relative risks of serum syndecan1 for high BVAS. BVAS, Birmingham vasculitis activity score; CI, confidence interval; RR, relative risk

Receiver operating tertile of BVAS, ROC) curve analysis revealed that the area under the curve (AUC of serum syndecan1 at diagnosis for the highest tertile of BVAS at diagnosis was statistically significant (0.864, 95% confidence interval 0.775, 0.953). The optimal cut-off of serum syndecan1 at diagnosis was calculated as the maximised summation of the sensitivity (70.4%) and specificity (90.4%) and was set as 76.1 ng/mL. When the patients were divided into two groups according to this cut-off, the highest tertile of BVAS at diagnosis was identified more often in patients with serum syndecan1 ≥ 76.1 ng/mL at diagnosis than those without (79.2% vs. 14.5%, *P* < 0.001). Furthermore, patients with serum syndecan1 ≥ 76.1 ng/mL at diagnosis showed a significantly higher risk for the highest tertile of BVAS at diagnosis than those without (RR 22.325, 95% CI 6.474, 76.985) (Fig. 1A).

Next, as for the upper half of BVAS, the optimal cut-off of serum syndecan1 was determined as 60.0 ng/mL (sensitivity, 64.4%; specificity, 79.4%) using the ROC curve analysis (AUC 0.752, 95% CI 0.647, 0.858). When the patients were divided into two groups according to this cut-off, patients with serum syndecan1 ≥ 60.0 ng/mL at diagnosis exhibited the upper half of BVAS at diagnosis more frequently than those without (80.6% vs. 37.2%, *P* < 0.001). Additionally, those with serum syndecan1 ≥ 60.0 ng/mL at diagnosis also showed a significantly higher risk for the upper half of BVAS at diagnosis than those without (RR 6.991, 95% CI 2.493, 19.608) (Fig. 1B).

### Relative risks of cut-off of serum syndecan1 at diagnosis for all-cause mortality during follow-up

**Fig. 2:**
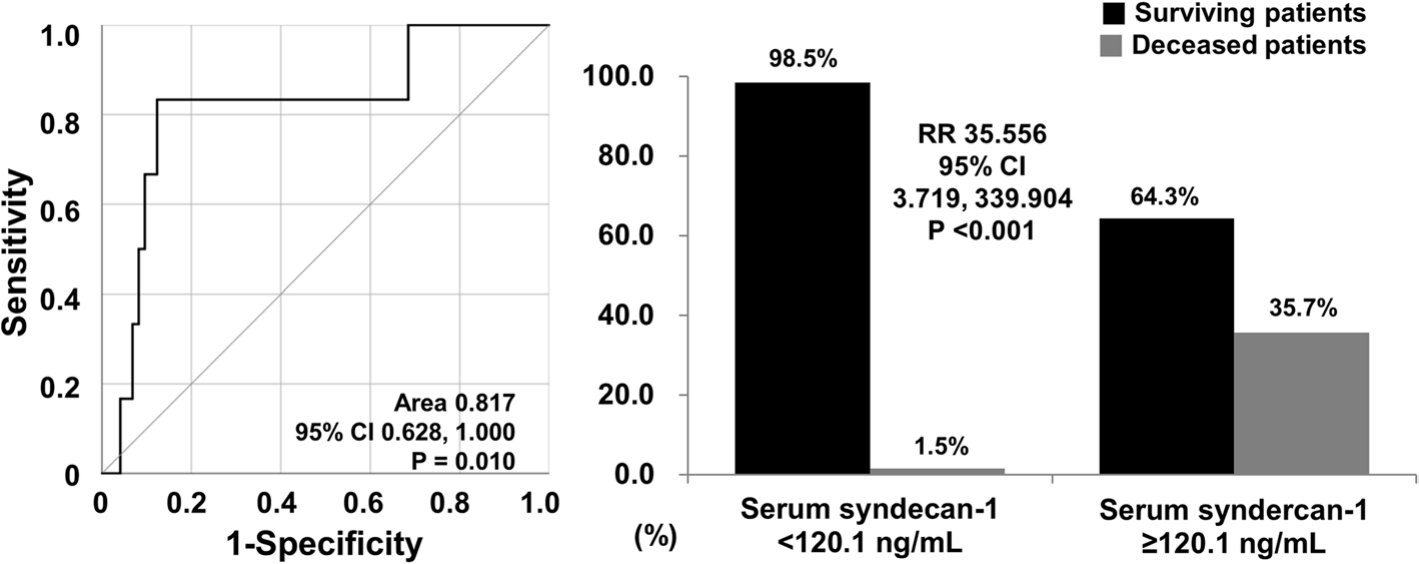
Optimal cut-off and relative risks of serum syndecan1 for all-cause mortality. CI: confidence interval; RR: relative risk

On the other hand, among the two poor outcomes of AAV during follow-up, the ROC curve analysis unveiled that the AUC of serum syndecan1 at diagnosis for all-cause mortality during follow-up was significant (AUC 0.817, 95% CI 0.628, 1.000). When the optimal cutoff of serum syndecan1 at diagnosis for all-cause mortality during follow-up was set at 120.1 ng/mL, the sensitivity and specificity were 83.3% and 87.7%, respectively. When patients were divided into two groups according to this cut-off, all-cause mortality during follow-up was found more often in patients with serum syndecan1 ≥ 120.1 ng/mL at diagnosis than those without (35.7% vs. 1.5%, *P* < 0.001). Moreover, patients with serum syndecan1 ≥ 120.1 ng/mL at diagnosis had a significantly higher risk for death than those without (RR 35.556, 95% CI 3.719, 339.904) (Fig. 2).

### Cumulative survival rate

**Fig. 3:**
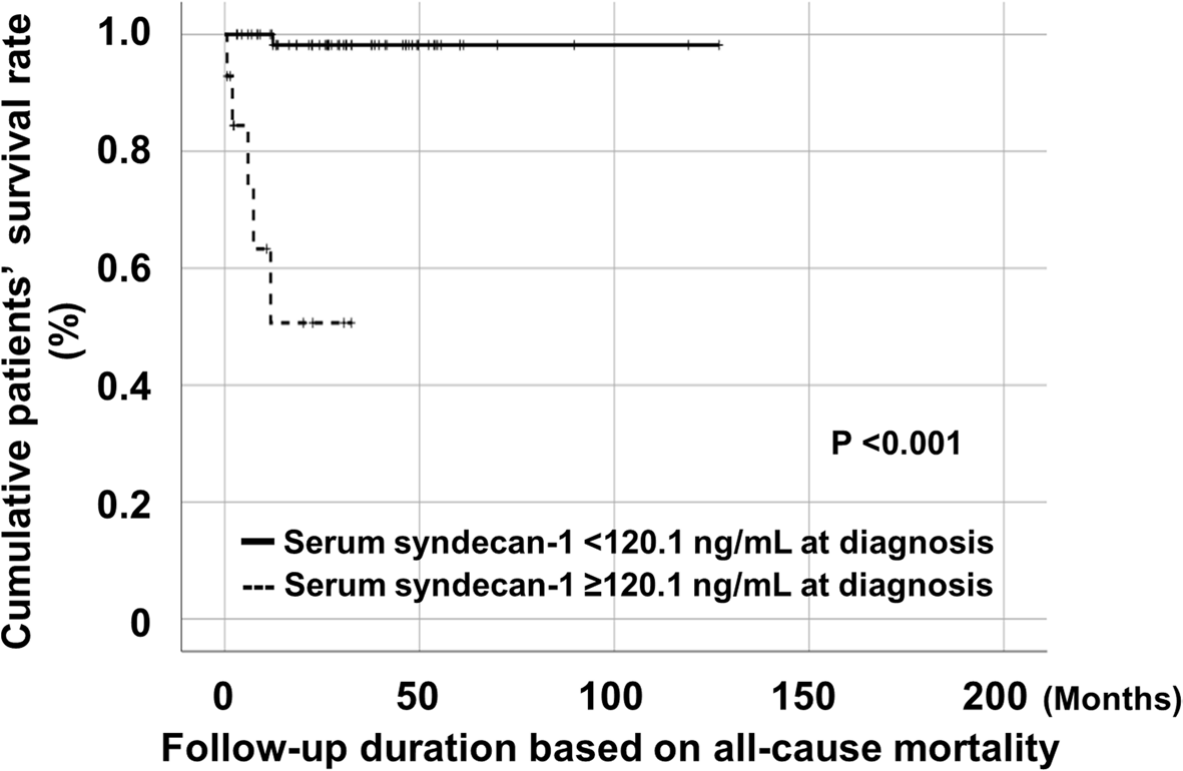
Comparison of cumulative survival rates

Patients with serum syndecan1 ≥ 120.1 ng/mL at diagnosis exhibited a significantly lower cumulative patient survival rate during follow-up than those with serum syndecan1 < 120.1 ng/mL at diagnosis (*P* < 0.001) (Fig. 3).

### Cox analyses

In the univariate Cox proportional analysis, the BVAS at diagnosis was not significantly associated with all-cause mortality during follow-up. Meanwhile, serum syndecans1 ≥ 120.1 ng/mL at diagnosis (HR 42.273), along with SF-36 PCS (HR 0.943), VDI (HR 1.591), dyslipidaemia (HR 11.068), white blood cell count (HR 1.130), haemoglobin (HR 0.604), and serum albumin (HR 0.152) at diagnosis were significantly associated with all-cause mortality during follow-up. However, in the multivariable Cox analysis of variables with statistical significance in the univariate analysis, none were independently associated with all-cause mortality during follow-up. Nevertheless, both dyslipidaemia (HR 9.928, 95% CI 1.000, 98.552, *P* = 0.005) and serum syndecans-1 ≥ 120.1 ng/mL (HR 59.822, 95% CI 0.611, 5,860.343, *P* = 0.080) at diagnosis exhibited the possibility of the independent association with all-cause mortality during follow-up in patients with AAV (Table 3).

**Table 3:** Cox hazards model analyses of variables at diagnosis for all-cause mortality during follow-up in AAV patients

Variables	Univariable			Multivariable		
	HR	95% CI	*P* value	HR	95% CI	*P* value
Age (years)	1.097	0.997, 1.207	0.057			
Male sex (N, (%))	2.933	0.537, 16.015	0.214			
Ex-smoker (N, (%))	0.047	0.000, 17,394,810.00	0.762			
BMI (kg/m^2^)	1.177	0.955, 1.450	0.126			
MPO-ANCA (or P-ANCA) positivity	4.573	0.533, 39.232	0.166			
PR3-ANCA (or C-ANCA) positivity	0.038	0.000, 552.366	0.504			
BVAS	1.076	0.994, 1.165	0.070			
FFS	2.178	0.842, 5.634	0.108			
SF-36 PCS	0.943	0.900, 0.988	0.013	0.998	0.933, 1.069	0.965
SF-36 MCS	0.972	0.935, 1.010	0.141			
VDI	1.591	1.069, 2.369	0.022	1.208	0.652, 2.239	0.548
T2DM	4.036	0.814, 20.008	0.088			
Hypertension	1.149	0.210, 6.273	0.873			
Dyslipidaemia	11.068	2.022, 60.570	0.006	9.928	1.000, 98.552	0.050
ESR (mm/hr)	1.017	0.995, 1.039	0.123			
CRP (mg/L)	1.016	1.000, 1.033	0.052			
White blood cell count (/mm^3^)	1.130	1.021, 1.250	0.018	1.228	0.919, 1.641	0.166
Haemoglobin (g/dL)	0.604	0.389, 0.939	0.025	1.287	0.467, 3.548	0.626
Platelet count (× 1000/mm^3^)	1.002	0.998, 1.006	0.376			
Blood urea nitrogen (mg/dL)	1.023	0.987, 1.061	0.210			
Serum creatinine (mg/dL)	1.258	0.847, 1.867	0.255			
Total protein (g/dL)	0.389	0.150, 1.011	0.053			
Serum albumin (g/dL)	0.152	0.047, 0.490	0.002	1.869	0.092, 38.111	0.684
C3 (mg/dL)	1.014	0.978, 1.052	0.448			
C4 (mg/dL)	0.957	0.845, 1.082	0.481			
Serum syndecans-1 ≥ 120.1 ng/mL	42.273	4.845, 368.842	0.001	59.822	0.611, 5,860.343	0.080

*SF-36* short form 36 health survey, *PCS* physical component summary, *AAV* ANCA-associated vasculitis, *ANCA* antineutrophil cytoplasmic antibody, *BMI* body mass index, *MPO* myeloperoxidase, *P* perinuclear, *PR3* proteinase 3, *C* cytoplasmic, *BVAS* Birmingham vasculitis activity score, *FFS* five-factor score, *T2DM* type 2 diabetes mellitus, *ESR* erythrocyte sedimentation rate, *CRP* C-reactive protein

## Discussion

In the present study, we investigated whether serum syndecan1 at diagnosis could reflect activity at diagnosis and predict poor outcomes during follow-up in patients with AAV and obtained several interesting findings. Firstly, serum syndecan1 at diagnosis exhibited significant correlations with AAV activity and functional status at diagnosis, as assessed by the BVAS, FFS, SF-36 PCS and MCS, and acute-phase reactants at di0agnosis, including ESR and CRP. Secondly, the ROC curve showed the significant AUCs of serum syndecan1 for the two concepts of high AAV activity at diagnosis: patients with serum syndecan1 ≥ 76.1 ng/mL at diagnosis, and those with serum syndecan1 ≥ 60.0 ng/mL at diagnosis showed significantly higher risks for the highest tertile and the upper half of BVAS at diagnosis than those without, respectively. Thirdly, patients with serum syndecan1 ≥ 120.1 ng/mL at diagnosis had a significantly higher risk for all-cause mortality during follow-up than those without, and further, exhibited a significantly lower cumulative patients’ survival rate than those without. Therefore, we conclude that in patients with AAV, serum syndecan1 at diagnosis may not only reflect AAV activity at diagnosis but may also be partially independently associated with all-cause mortality during follow-up.

Clinically, the most relevant immunological function of serum syndecan-1 in AAV pathogenesis likely involves its role in B cell differentiation and activation. This assumption appears to be supported by the role of circulating syndecan1 demonstrated in systemic lupus erythematosus and monoclonal gammopathy [3, 4]. The first assumption was that there may be a positive correlation between serum syndecan1 and B cell counts. To confirm this, we could follow the approach used in other studies by counting the activated B cells or total B cells using the fluorescence-activated cell sorting method and evaluated their alteration according to the circulating levels of syndecan1 [20]. Since our study is a retrospective analysis, we investigated the correlation between serum syndecan1 and lymphocyte count, however, found no significant correlation between them (*r* = − 0.127, *P* = 0.269) despite a positive correlation between serum syndecan1 and white blood cell count (*r* = 0.353, *P* = 0.001).

Secondly, a positive correlation may occur between serum syndecan1 and the total gamma globulin fraction. We indirectly evaluated the serum levels of paraproteins using the gamma gap which is defined as the difference between total serum protein and serum albumin (total serum protein – serum albumin) [21]. Serum syndecan1 was positively correlated with the gamma gap (*r* = 0.589, *P* < 0.001) along with serum albumin (*r* = − 0.451, *P* < 0.001), and further, the slope of the gamma gap was higher than that of serum albumin. In principle, patients with malignancies, including monoclonal gammopathy, were excluded from this AAV cohort at the time of enrolment, and the gamma gap indicated only a non-cancerous gamma-globulin fraction.

The third hypothesis is that there may be a positive correlation between serum syndecan1 and ANCA titres. We found that serum syndecan1 was significantly correlated with MPO-ANCA titres (*r* = 0.431, *P* < 0.001) among 79 patients though not with PR3-ANCA (*r* = − 0.039, *P* = 0.731). Additionally, we found that patients with MPO-ANCA (or P-ANCA) positive exhibited a significantly higher serum syndecan1 than those without (63.2 ng/mL vs. 41.8 ng/mL, *P* = 0.015). Collectively, although the correlation between serum syndecan1 and the level of B cell activation has not been clearly identified, the following hypothesis can be proposed: serum syndecan1 could indicate the serum levels of gamma-globulin production in B cells and further estimate MPO-ANCA titres at diagnosis. Therefore, this hypothesis suggests that serum syndecan1 may have the potential to reflect AAV activity at diagnosis by associating with MPO-ANCA (or P-ANCA)-specific clinical manifestations of BVAS.

The relative risk and cumulative survival rate of serum syndecan1 at diagnosis showed the possibility of an independent association with all-cause mortality during follow-up in patients with AAV. Additionally, in the univariate Cox analysis, the BVAS at diagnosis also tended to be associated with all-cause mortality (Table 3). Deceased patients had a significantly higher median serum syndecan1 than that of surviving patients (157.1 ng/mL vs. 48.2 ng/mL, *P* = 0.010). Serum syndecan1 may affect the occurrence of initial chest and renal manifestations of AAV, which may contribute to an increased rate of all-cause mortality in patients with AAV [22]. When comparing the total scores of the nine systemic items of the BVAS between surviving and deceased patients, deceased patients had a significantly higher median total score for chest manifestations than that of the surviving patients (4.0 vs. 2.0, *P* = 0.023). Furthermore, deceased patients showed a tendency for an increased median total score of renal manifestations compared with surviving patients (12.0 vs. 0, *P* = 0.060).

Additionally, we speculated whether serum syndecan1 at diagnosis might be associated with all-cause mortality during follow-up by enhancing the production of gamma-globulin in B cells at diagnosis. Several studies have reported a link between gamma gap and increased mortality risk in various populations [23, 24]. No significant difference was observed in the gamma gap between deceased and surviving patients (2.9 g/dL vs. 2.6 g/dL, *P* = 0.251), and further, the gamma gap at diagnosis was not significantly associated with all-cause mortality during follow-up in the univariable Cox analysis (HR 1.638, 95% CI 0.720, 3.729). Therefore, the association of serum syndecan1 at diagnosis with all-cause mortality during follow-up may not be due to alterations in the production of gamma globulin in B cells.

The strength of the present study is that it is the first to demonstrate the clinical implications of serum syndecan1 measured at diagnosis in patients with AAV in estimating vasculitis activity at diagnosis and has the potential to predict all-cause mortality during follow-up.

Critical limitations of this study are the small number of patients and the retrospective study design, despite the use of clinical data from a prospective observational cohort of AAV. Furthermore, due to the small group size, it is challenging to conduct separate analyses for MPO-ANCA-associated and PR3-ANCA-associated vasculitis. This study was unable to show dynamic changes in the correlation between serum syndecan1 and various variables simultaneously because of the limitations of being a cross-sectional study that did not include continuous clinical data. In addition, because of the limited availability of peripheral blood mononuclear cells, it was not possible to evaluate activated B cells or the entire B cell population; therefore, only indirect evidence for this mechanism is presented. Nevertheless, we believe that this study has clinical importance as a pilot study investigating the role of serum syndecan1 in patients with AAV. We also expect that future prospective studies with more patients and serial clinical data, including the measurement of serum syndecan1 levels during treatment, disease relapse, and remission, will provide more dynamic and reliable information on the clinical implications of serum syndecan1 in AAV.

## Conclusion

This study is the first to demonstrate that serum syndecan1 at diagnosis may not only reflect AAV activity at diagnosis but may also be associated with all-cause mortality during follow-up.

## Supplementary Information

Supplementary Material 1. Supplementary figure 1. Serum syndecan1 levels for all patients with MPA, GPA, and EGPA.

## Data Availability

Not applicable

## Abbreviations

ANCA: Antineutrophil cytoplasmic antibody
AAV: ANCA-associated vasculitis
BVAS: Birmingham vasculitis activity score
FFS: Five-factor score
SF-36: 36-item short-form survey
PCS: Physical component summary
MCS: Mental component summary
VDI: Vasculitis damage index
ESR: Erythrocyte sedimentation rate
CRP: C-reactive protein
MPA: Microscopic polyangiitis
GPA: Granulomatosis with polyangiitis
EGPA: Eosinophilic GPA
MPO: Myeloperoxidase
PR3: Proteinase 3
AUC: Area under the curve
ROC: Receiver operator characteristic
RR: Relative risk
HR: Hazard ratio

## Data availability

No datasets were generated or analysed during the current study.

## References

[1] Zhang X, Zhao Y, Liu L, He Y. Syndecan-1: a novel diagnostic and therapeutic target in liver diseases. Curr Drug Targets. 2023;24(15):1155–65. 37957867 10.2174/0113894501250057231102061624

[2] Kunnathattil M, Rahul P, Skaria T. Soluble vascular endothelial glycocalyx proteoglycans as potential therapeutic targets in inflammatory diseases. Immunol Cell Biol. 2024;102(2):97–116. 37982607 10.1111/imcb.12712

[3] Liu L, Akkoyunlu M. Circulating CD138 enhances disease progression by augmenting autoreactive antibody production in a mouse model of systemic lupus erythematosus. J Biol Chem. 2021;297(3): 101053. 34364875 10.1016/j.jbc.2021.101053 PMC8405997

[4] Maisnar V, Tousková M, Tichý M, Krejsek J, Chrobák L, Voglová J, et al. The significance of soluble CD138 in diagnosis of monoclonal gammopathies. Neoplasma. 2006;53(1):26–9. 16416009

[5] Jia X, Zhu Z, Miao J, Zhang L, Li X, Bao Y, et al. Serum Syndecan-1 levels in patients with immunoglobulin A vasculitis in children. J Pediatr (Rio J). 2022;98(5):526–32. 35240047 10.1016/j.jped.2022.01.004 PMC9510791

[6] Jennette JC, Falk RJ, Bacon PA, Basu N, Cid MC, Ferrario F, et al. 2012 revised international chapel hill consensus conference nomenclature of vasculitides. Arthritis Rheum. 2013;65(1):1–11. 23045170 10.1002/art.37715

[7] Watts R, Lane S, Hanslik T, Hauser T, Hellmich B, Koldingsnes W, et al. Development and validation of a consensus methodology for the classification of the ANCA-associated vasculitides and polyarteritis nodosa for epidemiological studies. Ann Rheum Dis. 2007;66(2):222–7. 16901958 10.1136/ard.2006.054593 PMC1798520

[8] Suppiah R, Robson JC, Grayson PC, Ponte C, Craven A, Khalid S, et al. 2022 American College of Rheumatology/European Alliance of Associations for Rheumatology classification criteria for microscopic polyangiitis. Ann Rheum Dis. 2022;81(3):321–6. 35110332 10.1136/annrheumdis-2021-221796

[9] Robson JC, Grayson PC, Ponte C, Suppiah R, Craven A, Judge A, et al. 2022 American College of Rheumatology/European Alliance of Associations for Rheumatology classification criteria for granulomatosis with polyangiitis. Ann Rheum Dis. 2022;81(3):315–20. 35110333 10.1136/annrheumdis-2021-221795

[10] Grayson PC, Ponte C, Suppiah R, Robson JC, Craven A, Judge A, et al. 2022 American College of Rheumatology/European Alliance of Associations for Rheumatology classification criteria for eosinophilic granulomatosis with polyangiitis. Ann Rheum Dis. 2022;81(3):309–14. 35110334 10.1136/annrheumdis-2021-221794

[11] Kitching AR, Anders HJ, Basu N, Brouwer E, Gordon J, Jayne DR, et al. ANCA-associated vasculitis. Nat Rev Dis Primers. 2020;6(1):71. 32855422 10.1038/s41572-020-0204-y

[12] Han CW, Lee EJ, Iwaya T, Kataoka H, Kohzuki M. Development of the Korean version of short-form 36-item health survey: health related QOL of healthy elderly people and elderly patients in Korea. Tohoku J Exp Med. 2004;203(3):189–94. 15240928 10.1620/tjem.203.189

[13] Stinton LM, Bentow C, Mahler M, Norman GL, Eksteen B, Mason AL, et al. PR3-ANCA: a promising biomarker in primary sclerosing cholangitis (PSC). PLoS ONE. 2014;9(11): e112877. 25397578 10.1371/journal.pone.0112877 PMC4232573

[14] Harper L, Chin L, Daykin J, Allahabadia A, Heward J, Gough SC, et al. Propylthiouracil and carbimazole associated-antineutrophil cytoplasmic antibodies (ANCA) in patients with Graves’ disease. Clin Endocrinol (Oxf). 2004;60(6):671–5. 15163328 10.1111/j.1365-2265.2004.02029.x

[15] Bossuyt X, Cohen Tervaert JW, Arimura Y, Blockmans D, Flores-Suárez LF, Guillevin L, et al. Position paper: revised 2017 international consensus on testing of ANCAs in granulomatosis with polyangiitis and microscopic polyangiitis. Nat Rev Rheumatol. 2017;13(11):683–92. 28905856 10.1038/nrrheum.2017.140

[16] Mukhtyar C, Lee R, Brown D, Carruthers D, Dasgupta B, Dubey S, et al. Modification and validation of the birmingham vasculitis activity score (version 3). Ann Rheum Dis. 2009;68(12):1827–32. 19054820 10.1136/ard.2008.101279

[17] Guillevin L, Pagnoux C, Seror R, Mahr A, Mouthon L, Toumelin PL, et al. The five-factor score revisited: assessment of prognoses of systemic necrotizing vasculitides based on the French Vasculitis Study Group (FVSG) cohort. Med (Baltim). 2011;90(1):19–27. 10.1097/MD.0b013e318205a4c6 21200183

[18] Flossmann O, Bacon P, de Groot K, Jayne D, Rasmussen N, Seo P, et al. Development of comprehensive disease assessment in systemic vasculitis. Ann Rheum Dis. 2007;66(3):283–92. 16728460 10.1136/ard.2005.051078 PMC1855994

[19] Murray CJ, Atkinson C, Bhalla K, Birbeck G, Burstein R, Chou D, et al. The state of US health, 1990–2010: burden of diseases, injuries, and risk factors. JAMA. 2013;310(6):591–608. 23842577 10.1001/jama.2013.13805 PMC5436627

[20] Kalina T, Fišer K, Pérez-Andrés M, Kuzílková D, Cuenca M, Bartol SJW, et al. CD maps-dynamic profiling of CD1-CD100 surface expression on human leukocyte and lymphocyte subsets. Front Immunol. 2019;10: 2434. 31708916 10.3389/fimmu.2019.02434 PMC6820661

[21] Dupuis MM, Paul B, Loitsch G, Mathews P, Feinberg D, Barak I, et al. Gamma gap: a point-of-care test that correlates with disease burden and treatment response in multiple myeloma. JCO Oncol Pract. 2020;16(8):e751-757. 32240071 10.1200/JOP.19.00517 PMC7427420

[22] Sánchez Álamo B, Moi L, Bajema I, Faurschou M, Flossmann O, Hauser T, et al. Long-term outcomes and prognostic factors for survival of patients with ANCA-associated vasculitis. Nephrol Dial Transpl. 2023;38(7):1655–65. 10.1093/ndt/gfac320 36617233

[23] Juraschek SP, Moliterno AR, Checkley W, Miller ER 3. The gamma gap and all-cause mortality. PLoS ONE. 2015;10(12): e0143494. 26629820 10.1371/journal.pone.0143494 PMC4668045

[24] Loprinzi PD, Addoh O. The gamma gap and all-cause mortality risk: considerations of physical activity. Int J Clin Pract. 2016;70(7):625–9. 27292974 10.1111/ijcp.12817

