# Supplementary figures and images for: Serum syndecan1 has the potential to reflect activity at diagnosis and predict death during follow-up in patients with ANCA-associated vasculitis
Source: Arthritis Res Ther. 2024 Sep 20;26:166. doi: 10.1186/s13075-024-03393-8 (PMC11414236; doi:10.1186/s13075-024-03393-8)

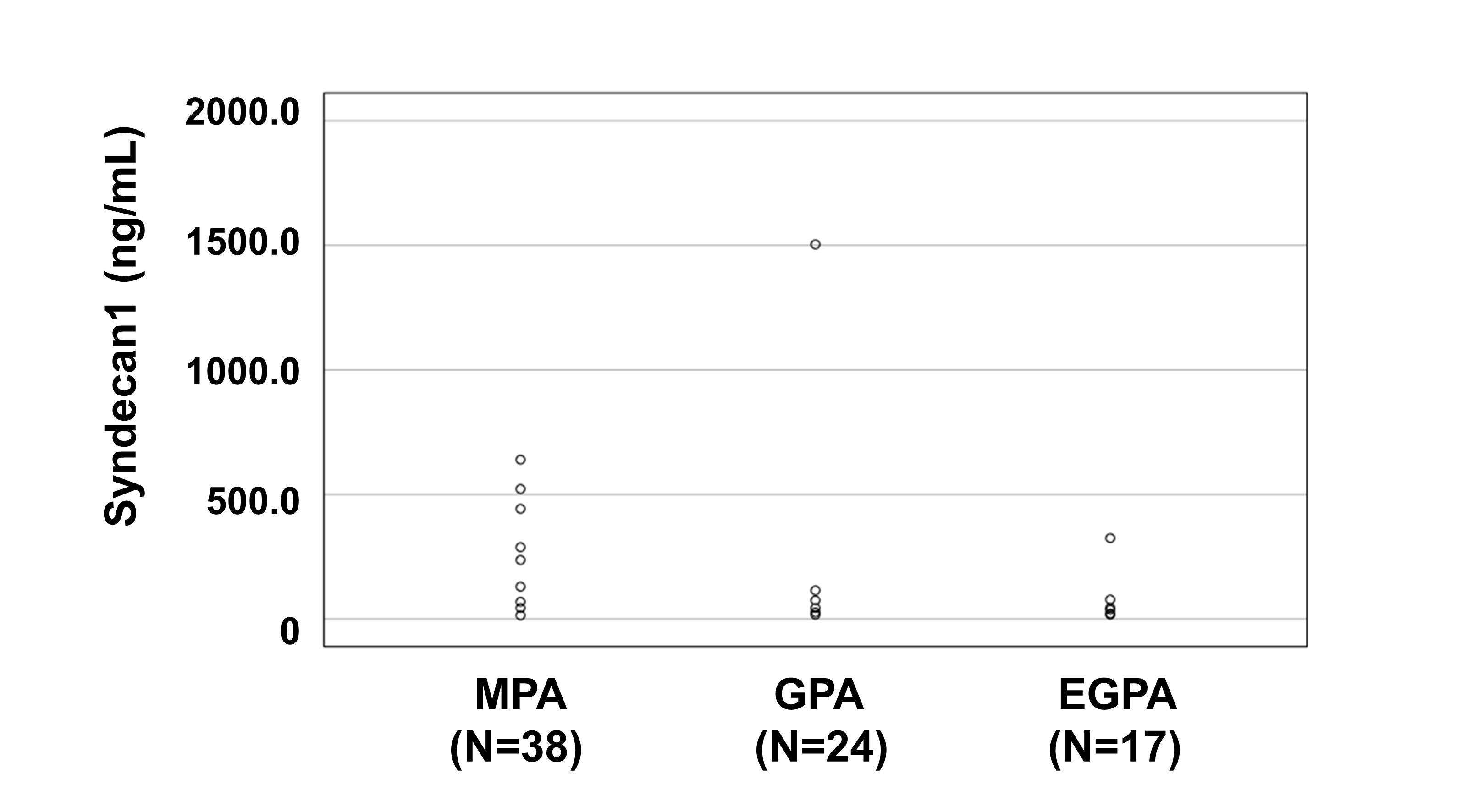

Supplement: Supplementary file 1 — Supplementary Material 1. Supplementary figure 1. Serum syndecan1 levels for all patients with MPA, GPA, and EGPA. [file 13075_2024_3393_MOESM1_ESM.tif]
